# Primary Vaccination with Low Dose Live Dengue 1 Virus Generates a Proinflammatory, Multifunctional T Cell Response in Humans

**DOI:** 10.1371/journal.pntd.0001742

**Published:** 2012-07-17

**Authors:** Janet C. Lindow, Nathan Borochoff-Porte, Anna P. Durbin, Stephen S. Whitehead, Kelly A. Fimlaid, Janice Y. Bunn, Beth D. Kirkpatrick

**Affiliations:** 1 Vaccine Testing Center and Unit of Infectious Diseases, Department of Medicine, University of Vermont College of Medicine, Burlington, Vermont, United States of America; 2 Johns Hopkins Bloomberg School of Public Health, Baltimore, Maryland, United States of America; 3 Laboratory of Infectious Diseases, National Institute of Allergy and Infectious Diseases, National Institutes of Health, Bethesda, Maryland, United States of America; 4 University of Vermont College of Mathematics, Burlington, Vermont, United States of America; Pediatric Dengue Vaccine Initiative, United States of America

## Abstract

The four dengue virus serotypes (DENV-1–DENV-4) have a large impact on global health, causing 50–100 million cases of dengue fever annually. Herein, we describe the first kinetic T cell response to a low-dose DENV-1 vaccination study (10 PFU) in humans. Using flow cytometry, we found that proinflammatory cytokines, IFNγ, TNFα, and IL-2, were generated by DENV-1-specific CD4^+^ cells 21 days post-DENV-1 exposure, and their production continued through the latest time-point, day 42 (*p*<0.0001 for all cytokines). No statistically significant changes were observed at any time-points for IL-10 (*p* = 0.19), a regulatory cytokine, indicating that the response to DENV-1 was primarily proinflammatory in nature. We also observed little T cell cross-reactivity to the other 3 DENV serotypes. The percentage of multifunctional T cells (T cells making ≥2 cytokines simultaneously) increased with time post-DENV-1 exposure (*p*<0.0001). The presence of multifunctional T cells together with neutralizing antibody data suggest that the immune response generated to the vaccine may be protective. This work provides an initial framework for defining primary T cell responses to each DENV serotype and will enhance the evaluation of a tetravalent DENV vaccine.

## Author Summary

40% of the world's population is at risk for developing dengue fever, an acute febrile illness caused by the 4 serotypes of dengue viruses (DENV). Though most of the 50–100 million annual DENV infections resolve without medical intervention, approximately 500,000 cases are severe and require hospitalization. Supportive care is currently the only available treatment for dengue disease. As a result, DENV infections cause strain on healthcare systems and economic burden in endemic countries. Much of the research in the dengue field has focused on understanding the mechanism of severe dengue disease. To better understand human adaptive immune responses to asymptomatic or mild DENV infections, we used longitudinal specimens collected following low dose vaccination with a live DENV-1 candidate vaccine. We found that CD4^+^ T cells made the proinflammatory cytokines, IFNγ, TNFα and IL-2, 3 weeks following exposure to DENV-1. IFNγ and TNFα production continued for 6 weeks post-vaccination, our final time-point. T cell responses were predominantly multifunctional: T cells produced ≥2 cytokines simultaneously. Lastly, we observed little cross-reactivity in T cell responses. This work helps establish the kinetics and characteristics of a primary adaptive immune response to DENV and aids in the development of a tetravalent vaccine against DENV.

## Introduction

Infections caused by the 4 serotypes of dengue virus (DENV-1- DENV-4) are significantly contributing to the increasing burden of vector-borne diseases globally [1]. Approximately 2.5 billion individuals living in >100 countries are currently at risk for acquiring DENV infection [1], [2]. The 4 DENV serotypes are endemic throughout the world's subtropical and tropical regions, especially in Asia and Latin America. DENV is also a re-emerging threat to areas in which it was previously controlled, as exemplified by recent 2009–2010 outbreaks in Florida [3]. Clinical disease ranges from the debilitating, but generally self-limited, dengue fever (DF) to life-threatening severe dengue disease which includes dengue hemorrhagic fever and dengue shock syndrome (DHF and DSS) [4].

Secondary infections, caused by a subsequent DENV infection in children and adults with a different DENV serotype than the primary infection, are associated with increased risk of severe disease (DHF/DSS) and are prevalent in endemic regions [5], [6]. Consequently, most studies have focused primarily on secondary DENV infections and comparisons of DF and severe disease rather than immune responses to primary infections. As safe candidate DENV vaccines are developed and introduced into naïve and endemic populations, a detailed understanding of the human immune response to both primary and secondary DENV infections is a necessary component of this effort. In particular, distinction is needed between post-infection immune responses which are beneficial or protective (including a correlate of protection) versus those leading to immunopathogenesis and DHF/DSS.

Two separate, but not mutually exclusive, hypotheses have been proposed to explain the immunopathogenesis of severe dengue disease: antibody-dependent enhancement (ADE) and cross-reactive memory T cells [7], [8], [9]. In the ADE hypothesis, cross-reactive antibodies from a prior infection increase the uptake of DENV by Fc-receptor-bearing cells but are incapable of fully neutralizing the virus [7]. Increased viral loads then trigger host inflammatory cascades and increased vascular permeability resulting in DHF/DSS. Notably, higher viral loads are correlated with severe dengue disease [10], [11]. The second hypothesis postulates that cross-reactive memory T cells from a previous DENV infection are preferentially activated over naïve T cells, producing an altered cytokine response that leads to a cytokine storm and ultimately ends in plasma leakage and DHF/DSS [12].

Complicating this picture is the observation that the 4 serotypes of DENV appear to be associated with distinct clinical and epidemiologic patterns, suggesting that each serotype varies in virulence and immunopathogenesis [13], [14], [15], [16], [17], [18], [19], [20]. The sequence of infection may also be important. In Cuba, for example, DENV-1 primary infections that were followed by DENV-2 or DENV-3 infections were highly associated with DSS [15], [16], [17]. Based on these observations, the primary immune responses may affect subsequent immune responses to different DENV serotypes. Thus, in order to understand DENV immunopathogenesis, we must understand the primary immune response to each DENV serotype.

Infection with live, attenuated monovalent DENV viruses offers a unique opportunity to define the precise kinetics of viral events for a known dose, serotype and genotype of DENV in a Flavivirus-naïve population that is not achievable in a natural setting. In natural DENV infections, evaluation of immune responses is complicated by an unknown date of infection and inoculum size. Prior DENV exposure history is often unknown or difficult to discern. Asymptomatic DENV infections are very difficult to capture. Furthermore, in endemic regions, multiple DENV infections are common making it impossible to study the precise effects of each serotype. Live monovalent DENV vaccines can be used as a model for asymptomatic or very mild DENV infection. Using longitudinal specimens from DENV-1 low dose vaccinees, we sought to better define the kinetics of the host cellular response and to evaluate the importance of serotype cross-reactivity. Our results suggest that multifunctional T cells that produce proinflammatory cytokines may facilitate control of DENV replication or clearance. Taken together, our data contribute to the understanding of the immune components that lead to recovery and protection from DENV infection.

## Materials and Methods

### Reagents

Fetal bovine serum (Atlanta Biologicals, Lawrenceville, GA); ACCUSPIN with Histopaque 1077 (A0561), Cell Freezing Medium (C6164), phorbol 12-myristate 13-acetate (PMA), β-mercaptoethanol and brefeldin A from Sigma-Aldrich, Inc. (St. Louis, MO); Hank's Balanced Salt Solution without Ca^2+^ or Mg^2+^ from HyClone (Logan, UT); all other reagents were purchased through Fisher unless specified. MEM GlutaMAX, phosphate buffered saline (PBS, pH 7.4), *Staphylococcus* enterotoxin B (SEB) [21], DNAase, RPMI 1640, and Live/Dead Blue were purchased from Gibco Invitrogen (Carlsbad, CA); and all antibodies and flow cytometry reagents were purchased from Becton Dickinson (Franklin Lakes, NJ).

### Ethics statement

Informed consent was obtained in writing from all subjects, and all work with human subjects was reviewed and approved by the University of Vermont Committees on Human Research prior to initiation of clinical studies.

### Clinical specimens from healthy volunteers

Peripheral blood mononuclear cells (PBMCs) and sera were collected from healthy adults participating in a phase I trial of a live, attenuated DENV-1 candidate vaccine. All volunteers had been screened by plaque reduction neutralizing titer assay (PRNT) and were found to be naïve to DENV, West Nile, St. Louis encephalitis, yellow fever virus, and Japanese encephalitis virus exposure. Volunteers were vaccinated with a single, subcutaneous 10 plaque forming units (PFU) dose of the live, attenuated dengue serotype 1 vaccine (DEN1Δ30). Vaccine dosage of 10 PFU was confirmed by titering vaccine virus immediately following administration of vaccine. Vaccine construct, as well as standardized assays to measure neutralizing antibody responses and viremia post-vaccination were performed as described [22], [23]. Table 1 describes the clinical signs, symptoms and PRNT_60_ for the volunteers in this study as previously reported (Lindow, manuscript in preparation). PBMCs were separated within 2 h of blood draw using the ACCUSPIN System (Histopaque 1077). Following gradient separation, cells were washed three times with Hank's Balanced Salt Solution without Ca^2+^ or Mg^2+^, and cryopreserved in Cell Freezing Media (Sigma) in liquid nitrogen. Cells from a total of 12 vaccinated volunteers were used in this study.

**Table 1:** DENV-1 vaccinees display a variety of clinical signs, symptoms and immunologic data.

Subject^a^	Viremia^b^	Rash	Neutropenia	Anemia	D28 PRNT_60_ c	D42 PRNT_60_ c
**02**	-	-	-	D8-9	91	192
**03**	D10-14	D14-28	-	-	232	41
**04**	D12	-	D14-16		273	101
**05**	D12	-	-	-	130	46
**06**	D12-16	-	-	-	56	59
**07**	D12-16	-	-	-	62	96
**08**	-	D14-21	-	-	58	167
**09**	-	-	-	-	18	55
**10**	D14-16	-	-	-	27	69
**12**	-	-	-	-	32	41
**14**	-	-	-		114	111
**18**	-	-	-	-	92	38
**Placebo 1**	-	-	-	-	<5	<5
**Placebo 2**	-	-	-	-	<5	<5

a Historical data as described in (Lindow, unpublished).

b Viremia was 0.5 log_10_/ml in serum on the days indicated as previously described (Lindow, unpublished).

c PRNT_60_ indicates the antibody titer required to neutralize 60% of wild type DENV-1 virus relative to a no serum control.

### Cell culture and DENV antigen preparation

Vero cells (WHO, a gift from S. Whitehead) were maintained using standard methods. Briefly, Vero cells were grown in MEM GlutaMAX (Minimum Essential Medium with glutamine, Invitrogen) plus 2% fetal bovine serum (FBS) at 37°C, 5% CO_2_. Inactivated DENV-infected Vero cell antigen was prepared as described previously [24]. Uninfected Vero cells were treated identically and used as negative controls for all experiments. Vero cells (85–90% confluent) were infected with DENV-1 (rDEN1 WP), DENV-2 (NCG Prototype), DENV-3 (Sleman/78) or DENV-4 (rDEN4) at an MOI = 0.01–0.25 and cultured in MEM GlutaMAX+2% FBS until >50% of the Vero cells were visibly cytopathic. Monolayers were harvested and the supernatant cleared by 6 min of centrifugation (1350*xg*) at 4°C. Cells were washed 2 times with phosphate buffered saline (PBS) (pH 7.4). The pellet was resuspended in PBS, fixed for 15 min with 0.025% gluteraldehyde at 4°C, washed 3 times with PBS, and resuspended in RPMI 1640+L-glutamine+10% FBS at 1/40 the original volume. Antigen preps were then sonicated on ice using a Fisher Scientific Sonic Dismembrator Model 100 (6, 30 s cycles at 60% power). Preps were centrifuged for 10 min at 4°C at 1350*x*g, the supernatant was aliquotted, and frozen at −80°C. Antigen was thawed immediately before use. Antigen was titrated for reactivity on DEN1Δ30 vaccinee samples. Briefly, each DENV antigen and corresponding Vero prep were tested on PBMCs specific to each DENV serotype at 1∶10, 1∶20 and 1∶40. DENV1 was also tested at 1∶100. The dilution resulting in the highest DENV-specific stimulation relative to Vero background was selected as the optimum dilution (data not shown). Time courses were also performed to determine the optimum stimulation conditions similar to those previously published [25]. Similar preparations were made for DENV-2 (NGC Prototype), DENV-3 (Sleman/78) and DENV-4 (rDEN4, S. Whitehead). A single preparation of antigen was used for all assays.

### Multiparameter flow cytometry

All assays were performed on a BD LSR II 4 laser flow cytometer. Thawed PBMCs were washed twice in 37°C cRPMI-10 (RPMI 1640+10% FBS)+2.7 U DNase/10^6^ cells/ml. Cells were resuspended at 5–10×10^6^/ml and rested for 6–18 h. Cells were washed once in warm c-RPMI-10. PBMCs were not used unless they had ≥86% viability following thaw and ≥90% viability following rest. The rest period allows dying cells to complete apoptosis prior to initiating the assay. The following was added to ∼1×10^6^ cells in wells containing 10 µg/ml brefeldin A (BFA) and anti-CD28/CD49d (1 µg/ml each) for 12 h: 1∶40 DENV-1 whole cell antigen, 1∶20 DENV-2 antigen, 1∶40 DENV-3 antigen, 1∶40 DENV-4 antigen, 1∶40 Vero antigen (negative control), 1∶20 Vero antigen (for DENV-2 only), or 3 µg/ml SEB (positive control). (As little antigen as possible was used as it autofluoresces in the FITC and APC channels, and had to be gated out using unstained cells treated with antigen.) After stimulation, cells were removed from culture plate for 10 min with 2 mM EDTA then washed twice with 1 ml PBS. Viability of cells was determined by staining with 1∶1000 Live/Dead Blue surface stain (Invitrogen) for 30 min at 4°C in the dark. Cells were washed in 1 ml PBS, fixed for 10 min at room temperature (RT) using BD FACS Lysing Solution, then washed twice with wash buffer 1 (WB1 = PBS+1% FBS+0.1% NaN_3_). Cells were then permeabilized for 10 min at RT using 250 µl BD FACS Permeabilization Solution 2. Cells were washed twice with WB2 (1∶10 BD Perm/Wash Buffer) followed by staining with the following cell surface markers and cytokines: CD3^+^ PerCP Cy5.5 (UCHT1), CD4^+^ V500 (RPA-T4), CD8^+^ V450 (RPA-T8), IFNγ Cy5.5 (B27), TNFα FITC (6401.1111), IL-2 PE (5344.111) and IL-10 APC (JES3-19F1). Stained cells were washed twice with WB2 and run the same day on a BD LSR II. Compensation was performed using beads prepared according to manufacturer's protocol (Becton Dickinson). Fluorescence minus one (FMO) experiments were run on multiple individuals as gating controls. 300,000 events were collected for a majority of time-points, with a minimum of 175,000 events for any time-point analyzed, and all with lymphocyte viabilities ≥90%.

### Data analysis

Data were analyzed using FlowJo version 9.3.1 (TreeStar) and SPICE v.5.22Beta software [26]. Boolean gating was used to determine simultaneous cytokine production in cells. For each of the cytokines, a positive response is defined as the difference between the DENV-antigen stimulated cells and the background (uninfected Vero control). The fold-increase of antigen-stimulated cells divided by Vero-stimulated cells (background) was statistically higher on days 21, 28, and 42 for IFNγ and TNFα (*p*<0.05) and days 21 and 28 for IL-2 (*p*<0.01). All wells produced ≥3 cytokines when stimulated with SEB. PMA-ionomycin stimulated cells produced all 4 of the analyzed cytokines. Increasing CD3^+^ and CD4^+^ gate sizes did not alter the results shown.

### Statistical analysis

Cytokine data was analyzed following background subtraction (the identical cell population treated with uninfected Vero prep) from the DENV stimulated sample. The fold increase of signal over background was also analyzed for all cytokines at all time-points. A single-group repeated measures analysis of variance was used to examine cytokines or multifunctional T cells produced at each time-point relative to pre-vaccination. Additionally, differences in multifunctional T cell production between those who did and did not develop viremia were tested using a two-group repeated measures analysis of variance. Repeated measures analyses were performed using SAS, version 9.2; Wilcoxon rank sum tests were computed using SPICE v.5.22Beta. *p* values≤0.05 were considered significant.

## Results

To evaluate cellular immune responses following infection with DENV-1, we collected peripheral blood mononuclear cells (PBMCs) at pre-vaccination and at weekly time points post-vaccination (days 8,14,21,28,42) from the 15 Flavivirus-naïve volunteers vaccinated with 10 plaque forming units (PFU) of the live, attenuated DENV-1 vaccine, rDEN1Δ30, and 2 placebo recipients. A full description of clinical outcomes is described elsewhere (J Lindow, manuscript in preparation). We observed low-level DEN1Δ30 viremia (0.5 log_10_/ml serum), starting at study day 10 post-vaccination, in 8/15 (53%) vaccinees, as determined by a standardized viral amplification assay. Two individuals experienced mild rash, and 14/15 volunteers (93%) seroconverted to DENV-1, as defined as ≥4-fold increase in PRNT_60_ (60% of virus is neutralized by antibodies) to wild type DENV-1 on post-vaccination days 28 or 42 compared to the pre-vaccination titer (day 0). We did not detect any other clinical signs or symptoms such as leukopenia or elevated liver enzyme levels in any of the volunteers. 12/15 subjects, 6 viremic and 6 non-viremic, along with 2 placebo recipients were randomly selected for phenotypic responses of T cell responses (Table 1). All 12 subjects seroconverted to DENV-1.

### Antigen-specific pro-inflammatory cytokines are produced by CD4^+^ T cells post-DENV-1 vaccination

To evaluate the kinetics of the T cell cytokine responses following DENV-1 primary infection, we stimulated PBMCs collected from a pre-vaccination time-point (day 0) and 5 post-vaccination time-points (days 8, 14, 21, 28, and 42) with either wild type DENV-1 antigen or a negative control antigen (Vero cells). The DENV-1 antigen contains structural and non-structural proteins and therefore allows an unbiased, global analysis of T cell responses. Using an 8-color flow cytometry panel, we then measured the number of CD3^+^ CD4^+^ or CD3^+^ CD8^+^ T cells producing cytokines that have been implicated in natural dengue disease: IFNγ, TNFα, IL2, and IL10 [27], [28], [29], [30]. Representative gating of CD4^+^ T cells from DENV-1-stimulated cells (Subject 03, day 28) is shown in **Figure S1**.

**Figure 1:**
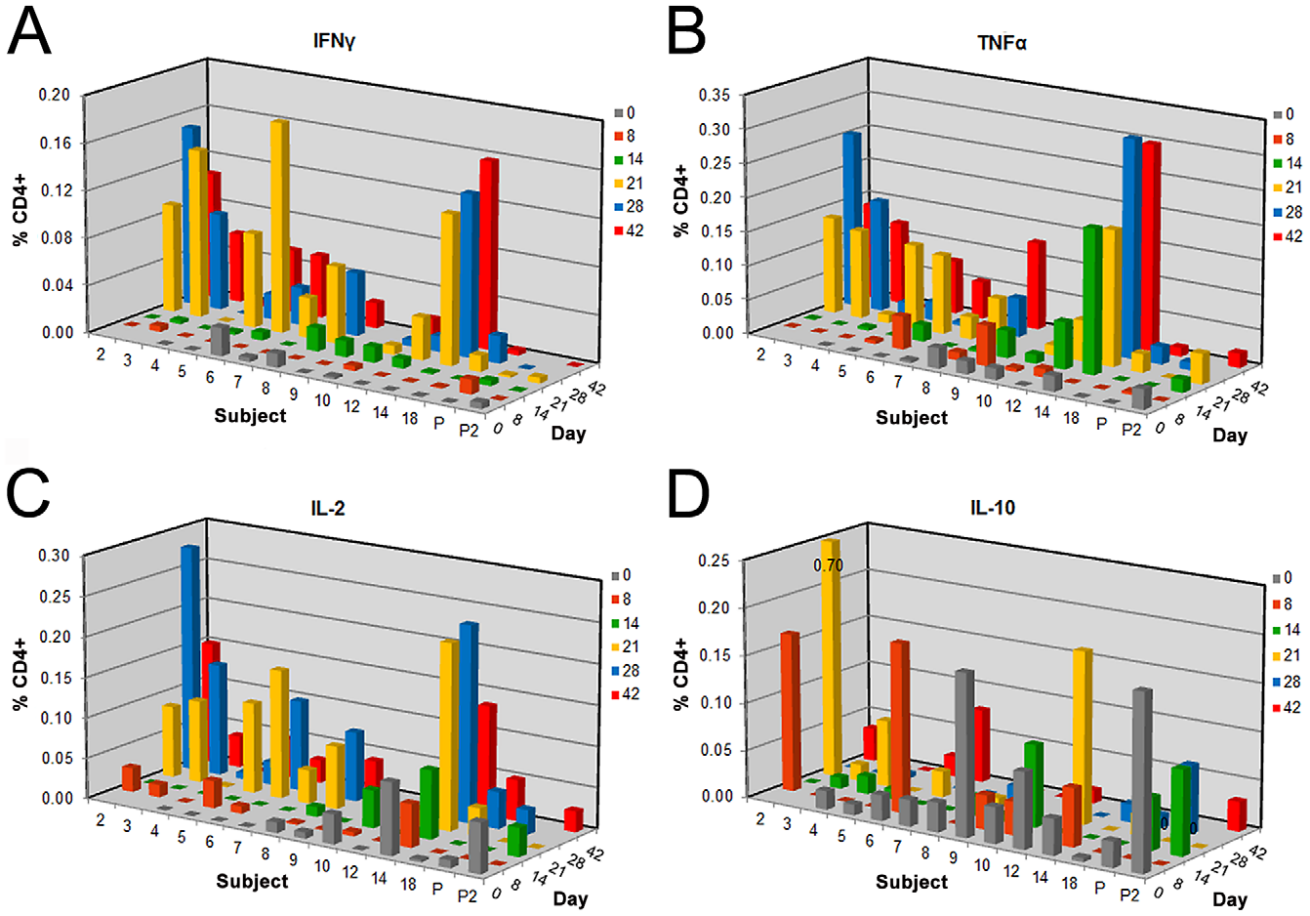
Proinflammatory cytokines are produced at specific times post-vaccination with live DENV-1. Cytokine profiles for 12 subjects vaccinated with 10 PFU of live monovalent DENV-1 and 2 placebo recipients. CD4^+^-dependent production of 4 cytokines are shown for pre-vaccination (day 0) and 5 post-vaccination time-points following *ex vivo* stimulation with homologous, wild type DENV-1 antigen: **A.** IFNγ, **B.** TNFα, **C.** IL-2 and **D.** IL-10. Responses shown are the percentage of cytokine positive T cells from DENV-1-stimulated PBMCs with the background percentage of cytokine-positive T cells in the negative control (Vero) subtracted. Clinical information for each subject is shown in **Table 1**. Significantly higher levels of IFNγ and TNFα were observed on days 21, 28 and 42 (*p*<0.05 for IFNγ and *p*<0.01 for TNFα), and on days 21 and 28 for IL-2 (*p*<0.01). For **A–C**, *p*<0.0001 for group by day differences demonstrating increased cytokines with time compared to day 0.

CD4^+^ T cell responses for pre- and post-vaccination time-points for DENV-1 vaccinees are summarized in **Figure 1**. All 3 proinflammatory cytokines, IFNγ, TNFα and IL-2, were produced by CD4^+^ T cells 3 weeks after DENV-1 infection (**Figure 1A–C**). More IFNγ^+^ CD4^+^ and TNFα^+^ CD4^+^ T cells were present on days 21, 28 and 42 relative to day 0 (pre-vaccination) (*p*<0.0001 for analysis of differences over time; *p*<0.05 and *p*<0.01 for days 21–42 versus day 0 for IFNγ and TNFα, respectively) (**Figure 1A and B**). Significantly more IL-2^+^ CD4^+^ T cells were also observed on days 21 and 28 relative to day 0 (*p*<0.0001 for differences over time; *p*<0.01 for days 21 and 28) (**Figure 1C**). The timing of these T cell responses occurred 7–11 days after the onset of detectable viremia based on the 6 viremic subjects tested. Of particular interest, we did not observe any significant trends in the production of IL-10, a regulatory cytokine (*p* = 0.19) (**Figure 1D**).

We observed little or no stimulation of CD8^+^ T cells using this DENV-1 antigen (data not shown). This is similar to what other groups have observed using this type of DENV antigen [22], [24].

### DENV-1 exposure results in a multifunctional T cell response

**Figure 2:**
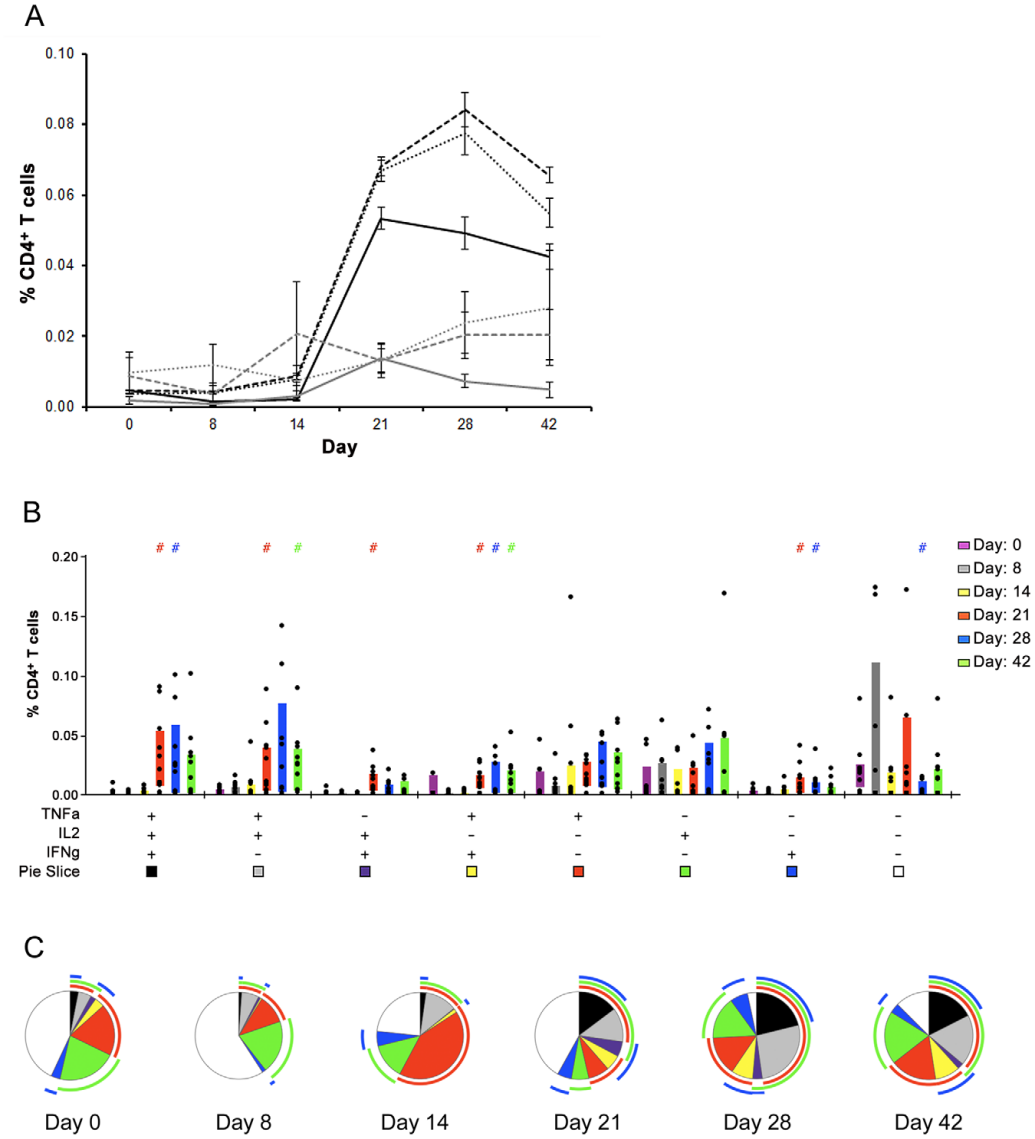
Multifunctional T cells are produced following exposure to 10 PFU of a DENV-1 monovalent vaccine. **A.** Distribution of cytokines produced by multifunctional or monofunctional T cells. The percent of multifunctional CD4^+^ T cells producing IFNγ (black line), TNFα (black dashes), and/or IL-2 (black dots) over time relative to the number of CD4^+^ T cells making a single cytokine: IFNγ (gray line), TNFα (gray dashes) or IL-2 (gray dots). Error bars represent standard error, n = 9–11 depending on time-point. **B.** IFNγ, IL2, and TNFα production in CD4^+^ T cells as a function of time and co-production. Each day is represented with a single color. Black dots correspond to the response from a single subject. Shaded bars represent the interquartile range. Line separates multifunctional T cells (left) from monofunctional T cells (right). A “+” or “−” indicates whether a particular cytokine (TNFα, IFNγ and/or IL-2) is present. **C.** Proportions of total CD4^+^ T cells producing IFNγ (blue), IL2 (green), TNFα (red), or different combinations of the three (colors indicated by boxes in **B**) are shown for 5 post-vaccination time-points (days 8, 14, 21, 28, and 42) relative to pre-vaccination (day 0) for 11 vaccinees. Overlaps in red, green, and/or blue arcs indicate T cells producing the correlated cytokines simultaneously. A “ #” denotes statistically a significant difference between a response on a post-vaccination day and day 0 (*p*<0.05 by Wilcoxon's rank sum test). There are statistically higher percentages of total multifunctional T cells on days 21, 28, and 42 (*p*<0.0001).

Multifunctional T cells (T cells making ≥2 cytokines simultaneously) have been correlated with both protection from disease and lessening of progression of disease for a variety of human pathogens including HIV, *Leishmania major*, and *Mycobacterium tuberculosis* [31], [32], [33], [34], [35]. We Boolean gated the CD4^+^ T cell cytokine responses to determine the breadth of the T cell response following DENV-1 infection. We observed that multifunctional T cells increased with time; on days 21–42 we observed a significant increase in the number of multifunctional T cells compared to days 0–14 (p<0.0001) (**Figure 2A and B**). To determine the abundance of multifunctional T cells relative to T cells producing only 1 of the 3 proinflammatory cytokines, we summed the total number of multifunctional T cells producing each of the cytokines for a particular day and divided the sum by the number of T cells making only that cytokine alone. We found that there were 3.8-fold, 5.1-fold, and 5.0-fold more multifunctional T cells making IFNγ^+^, TNFα^+^, and IL-2^+^, respectively, compared to single cytokine producing T cells (**Figure 2A**). We observed similar increases on days 28 and 42 (**Figure 2A**).

We next wanted to analyze each possible T cell phenotype (8 possible with 3 cytokines) to determine whether specific combinations of cytokines were produced at specific times post-DENV-1 infection. To this end, we plotted the percentage of CD4^+^ T cells producing a particular combination of cytokines for each of the 6 time-points (days 0, 8, 14, 21, 28, and 42) as shown in **Figure 2B**. This analysis revealed that there is a marked increase in IL-2^+^ IFNγ^+^ TNFα^+^ CD4^+^ T cells (triple producers) 3 to 6 weeks post-vaccination (days 21–42), with significant increases at days 21 and 28 relative to day 0 (*p* = 0.005 and *p* = 0.049, respectively) and day 42 approaching significant difference from day 0 (*p* = 0.055) (**Figure 2B**). While T cells simultaneously producing all 3 cytokines were relatively abundant, we also found significant numbers of CD4^+^ T cells making different combinations of 2 cytokines(IFNγ^+^ TNFα^+^, TNFα^+^ IL-2^+^, and IL-2^+^ IFNγ^+^) on days 21–42 relative to pre-vaccination (day 0) (*p*≤0.023) (**Figure 2B**).

To better understand how each of the 8 different T cell subsets changed over time, we generated pie charts for each day, with each slice representing the proportion of a specific T cell subset compared to the entire T cell population. The pie charts show that multifunctional T cell subsets increase as a function of time, emerging as early as day 14 (**Figure 2C**). Further, the pie charts show that T cells making only 1 cytokine (red, green, or blue) represent a smaller fraction of the total T cell population, especially at later time-points (days 21–42) (**Figure 2A and C**). Together these data show that the proinflammatory CD4^+^ T cell response to DENV-1 is largely multifunctional and lasts a minimum of 6 weeks post-infection.

### Fewer multifunctional T cells may be generated by subjects who develop viremia

**Figure 3:**
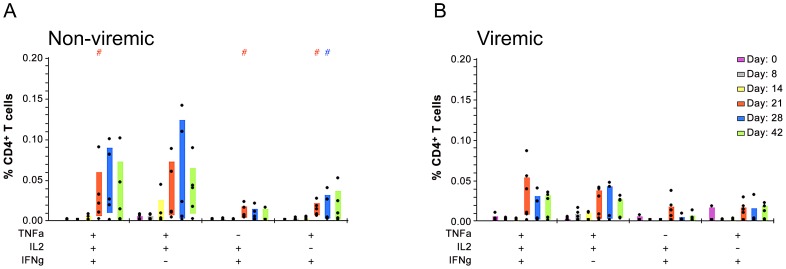
Non-viremic vaccinees may produce more multifunctional T cells than viremic volunteers. **A.** Multifunctional CD4^+^ T cells produced by non-viremic group. **B.** Multifunctional T cell profile for viremic group. # denotes statistically higher % CD4^+^ cells producing the cytokine profiles indicated compared to Day 0 (*p*<0.05 by Wilcoxon's rank sum test). Each day is represented by a separate color. A “+” or “−” indicates whether a particular cytokine (TNFα, IFNγ and/or IL-2) is present. Shaded bars denote interquartile ranges.

53% of DEN1Δ30 vaccinees developed low level viremia (0.5 log_10_ PFU/ml) 10–14 days post-infection. To test whether individuals who developed viremia had a delayed or less robust T cell response, we compared CD4^+^ T cell cytokine responses from the 6 individuals who developed viremia to 5 individuals who did not, at all time-points. We were not able to collect ≥250,000 events for 1 viremic subject past day 8 so we excluded the subject from this analysis. We found no significant differences in the pattern and/or magnitude of individual cytokines produced over time by CD4^+^ T cells between viremic and non-viremic subjects (*p*≥0.13 for each cytokine). However, we observed that on day 28, non-viremic subjects were trending toward having elevated multifunctional T cells relative to viremic subjects (*p* = 0.064) (**Figure 3A and B**).

### Low dose DENV-1 exposure results in minimal cross-reactivity to heterologous DENV antigens

**Figure 4:**
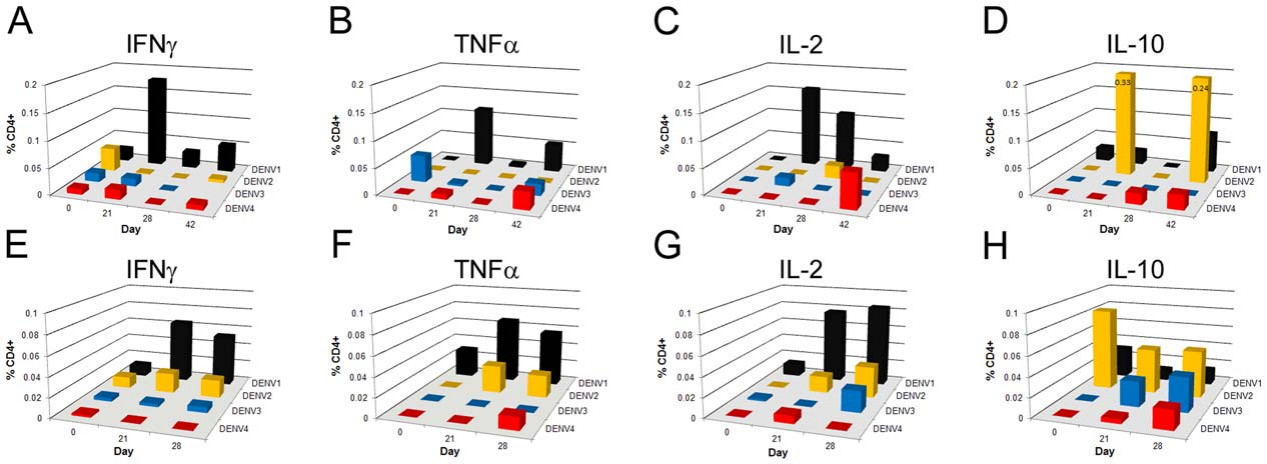
Little T cell crossreactivity when stimulated with heterologous DENV antigens. Cytokine production in CD4^+^ T cells for 2 subjects (Subject 06, viremic (**A–D**) and Subject 08, non-viremic (**E–H**)) in response to stimulation with homologous (DENV-1, black bars) or heterologous stimulation with DENV-2 (yellow), DENV-3 (blue) or DENV-4 (red). Shown are the % CD4^+^ T cells producing a particular cytokine at pre-vaccination (day 0) and post-vaccination time-points: IFNγ (**A, E**), TNFα (**B, F**), IL-2 (**C, G**), and IL-10 (**D, H**). Insufficient cells were available at day 42 for **E**, **F**, **G**, **H** for analysis.

One possible mechanism for increased disease severity upon exposure to a new, heterologous DENV serotype in a secondary infection is that memory T cells from a primary DENV infection are activated and produce an altered, low affinity response to the infecting DENV serotype that is insufficient to clear the new infection. To determine whether cross-reactive T cells were generated following DENV-1 infection, we stimulated PBMCs from 2 subjects with each of the 4 DENV serotypes separately (**Figure 4**). Fewer CD4^+^ T cells responded to heterologous antigens compared to DENV-1, thus, the cytokines produced were of lesser magnitude than the DENV-1 response, were observed at later time-points (day 28 or day 42) (**Figure 4**). Neither subjects' T cells produced IFNγ significantly above the day 0 value in response to heterologous DENV stimulation (**Figure 4 A, E**). Both subjects showed a very minor response to DENV-4 with production of TNFα at day 42 (Subject 1) or day 28 (Subject 2), and 1 subject responded to DENV-2 (Subject 2) by making small amounts of TNFα. Again, all responses were well below those observed for the homologous DENV-1 antigen. IL-2 was produced on day 42 in Subject 1 when stimulated with DENV-4 while Subject 2 responded similarly, though to a lesser extent, to DENV-2 and DENV-3 on day 28. Lastly, a robust IL-10 response was observed in Subject 1 when stimulated with DENV-2 on days 21 and 42, but not day 28. Subject 2 produced minor amounts of IL-10 when stimulated with DENV-3 or DENV-4. Together, these results show that the level of cross-reactivity in the T cell responses is relatively low, at least in comparison to the response to homologous DENV-1 antigen in a small number of subjects.

## Discussion

We demonstrate that a DENV-1-specific CD4^+^ T cell response with specific kinetics was elicited in Flavivirus-naïve, healthy adults following vaccination with a low dose (10 PFU) of live DEN1Δ30 virus. The observed cytokine response was proinflammatory; IFNγ, TNFα and/or IL-2 production became evident 3 weeks following vaccination. We did not detect significant amounts of IL-10, a regulatory cytokine, at any particular time post-vaccination. We also noted no or low levels of DENV serotype T cell cross-reactivity in a small number of subjects. Lastly, we found that a majority of the cytokines were produced by multifunctional T cells (≥2 cytokines produced simultaneously). As has been observed in other infectious diseases, the presence of multifunctional T cells following DENV-1 vaccination suggests that live, attenuated DENV vaccines safely and effectively prompt immune responses associated with control of infection and protection from re-infection.

In natural primary DENV infections, the precise timing of infection cannot be determined, but is generally 3–14 days prior to disease onset, and disease symptoms last approximately 1 week [36]. DENV-specific T cells have been observed 0–5 days prior to defervescence, when viremia levels were dropping or undetectable [9], [37]. In our vaccine platform, low level viremia (0.5 log_10_ pfu/ml serum) occurred 10–16 days after vaccination in 6 subjects, but DEN1Δ30 virus was undetectable in the other 5 subjects, though all seroconverted to DENV-1. Statistically significant CD4^+^ T cell responses to DEN1Δ30 vaccination were measurable 21 days following vaccination and pro-inflammatory cytokines remained elevated for at least 6 weeks, the latest time-point samples were available. In the 6 subjects with viremia, which occurred about 2 weeks post-vaccination, the T cell responses occurred later than expected and were only measurable 5–11 days after the onset of viremia. Similar kinetics of T cell responses were observed in studies in macaques: T cell responses were evident ∼20 days following the peak of viremia (days 3 to ≥11) in macaques inoculated with a high dose tetravalent DENV vaccine, DENVax [38]. Given these similarities, we suspect that the increased length of time required to elicit a measurable T cell response is a function of the attenuated DEN1Δ30 virus or inoculum size. The live-attenuated DEN1Δ30 vaccine candidate has decreased replication kinetics, which may slow the spread of the virus, resulting in lower antigenic loads and delayed or muted T cell responses.

The role of proinflammatory cytokines in the immunopathogenesis of DENV is a research area of intense interest. Proinflammatory cytokines such as TNFα, IFNγ, and IL-6 have been associated with severe secondary dengue disease in several studies [27], [28]. Far less is known about the role of these cytokines in primary disease, and in particular, mild or asymptomatic disease. In a study that included primary natural DENV infections, higher percentages of T cells (CD4^+^ and CD8^+^) produced IFNγ and/or TNFα in DHF versus DF patients during the acute phase of disease [39]. However, a recent study demonstrated that the pro-inflammatory cytokines, TNFα, IFNγ and IL-2, were produced by T cells (CD8^+^ and CD4^+^) at higher levels in individuals who subsequently developed subclinical secondary DENV infections versus symptomatic infections [40]. Human challenge studies conducted with under-attenuated DENV vaccines also correlated sustained levels of IFNγ produced by PBMCs with protection from re-challenge with the same viruses [41]. These studies indicate that the production of proinflammatory cytokines may be part of a continuum between asymptomatic infection and pathogenesis.

Understanding the characteristics of T cells, including multifunctional T cells, involved in protection from DENV is important for assessing vaccine immunogenicity, safety, and efficacy. In other disease models, extensive work has focused on the role of multifunctional T cells, both CD4^+^ and CD8^+^, and protection from disease or lessening of disease severity in a variety of infections: HIV, tuberculosis, malaria, leishmaniasis, influenza, and vaccinia [31], [32], [33], [34], [35], [42], [43], [44]. We observed significantly increased numbers of multifunctional CD4^+^ T cells by day 21 of infection, and these largely remained present through day 42. While we do not have data past 6 weeks to determine the duration of the multifunctional responses, our initial results parallel other models, and offer the first suggestion that multifunctional T cells may be associated with protective responses.

We observed a trend of more multifunctional T cells in non-viremic vaccinees relative to viremic vaccinees. While these data did not reach statistical significance because of the limited number of available subjects, they suggest that multifunctional CD4^+^ T cells may be indicators of individuals who are better able to control DENV infection, and therefore may have less severe clinical disease. This is consistent with findings from HIV studies: individuals with lower viral loads have larger percentages of multifunctional T cells, in particular IFNγ^+^ TNFα^+^ IL2^+^ (triple producers) or IFNγ^+^ IL-2^+^ (double producers) CD4^+^ T cells, while those who have higher viral loads and more severe disease progression produce greater numbers of IFNγ+ CD4^+^ T cells [45], [46], [47], [48]. However, HIV causes a chronic infection while DENV results in an acute infection. This difference might affect the nature of the T cell response. More work is clearly required to discover whether the correlation between levels of viremia for DENV and production of multifunctional T cells is a reason some individuals may develop more severe DENV disease. Of note, no significant differences in neutralizing antibody titers were observed between the viremic and nonviremic subjects or high (Subjects 2, 3, 5, 6, 8, 14) and low (Subjects 4, 7, 10, 12, 18) T cell responders; all groups generated robust antibody responses. Future work should also address whether memory T cell responses maintain specific multifunctional properties or reduce the number of cytokines produced, and whether memory T cell populations are correlated with neutralizing antibody outcome.

The role of IL-10 in DENV disease is still contradictory as it has been correlated with severe dengue disease, despite having multiple regulatory functions that aid in DENV clearance [30], [49]. In general, IL-10 is a regulatory cytokine, produced by CD4^+^ T cells, activated CD8^+^ T cells, and activated monocytes, that can reduce IFNγ-related antiviral effects [50], [51], [52], [53]. We detected little to no IL-10 production at each specific time-point suggesting that it is either not produced at the times sampled following DENV-1 inoculation or our flow assays did not capture the cell population producing it. The role of IL-10, if any, following natural DENV exposure or vaccination therefore requires further investigation.

Our work also contributes to the understanding of memory T cell cross-reactivity following DENV infections, which is thought to contribute to increased disease severity [8]. While we observed small increases in the number of cross-reactive CD4^+^ T cells producing TNFα, IL-2 or IL-10, especially on day 42, the magnitude of the responses were much lower than those observed for homologously stimulated T cells (stimulated with DENV-1 antigen). Similar to the rationale outlined above for delayed T cell responses, the low dose and/or attenuation of the infecting DENV-1 vaccine strain may decrease the antigenic load affecting the overall T cell response, including cross-reactivity. Higher viral loads, as seen in natural exposure, may be required to activate lower affinity T cells such as memory T cells, which cross-react to a different DENV serotype [8]. Alternatively, the difference between our data and other reports could be the result of the small subject numbers or differences in study populations. The issue of T cell cross-reactivity is important to clarify as it potentially affects dengue vaccine safety in Flavivirus-experienced populations. Future work will continue to address whether the dose of virus affects the development of cross-reactive T cell responses and whether cross-reactivity varies amongst populations.

Although CD4^+^ T cells play an important role in resolving of DENV infections, CD8^+^ T cells are active participants in clearing DENV infections [54], [55], [56], [57]. We likely did not observe robust CD8^+^ T cell responses because the inactivated DENV antigens we used are not ideal antigens for CD8^+^ stimulation from frozen PBMCs which are not able to process full-length antigens efficiently [40]. Low CD8^+^ responses have been observed using this type of antigen by other groups [24], [25]. Future work using specific DENV Class I peptides will help define the role of CD8^+^ in the overall T cell response to monovalent live vaccines.

Longitudinal DENV vaccine specimens provide a unique opportunity to determine T cell profiles in primary, mild DENV infections in a controlled, clinical setting. Further analysis using these and other specimens from DENV monovalent vaccine clinical trials will enable us to determine whether there are differences among serotypes, since each DENV serotype may stimulate a unique cytokine production pattern in T cells. We can also test the effect of antigenic load on the immune response using specimens from different doses of the same vaccines. These are nearly impossible goals to achieve in natural settings as the time of infection and infectious dose cannot be determined, and sometimes even the infecting serotype is difficult to determine due to cross-reactive immune responses. To best determine the role of T cell responses in protection, future work will need to compare specimens from controlled settings to field-setting specimens from individuals with known clinical outcomes. A detailed understanding of a protective versus aberrant immune response following DENV infection will greatly enhance the development and evaluation of an efficacious tetravalent DENV vaccine.

## Supporting Information

Figure S1 **CD4^+^ T cells respond to DENV-1 antigen.** Gating scheme and raw representative data of PBMCs stimulated with DENV-1 antigen for Subject 03 on post-vaccination day 28. Cells are initially gated on singlets followed by the lymphocyte population. CD3^+^ are gated from the lymphocyte population after selecting for live cells. CD4^+^ and CD8^+^ are separated from the CD3^+^ population. CD4^+^ CD8^−^ cells are gated for IFNγ, TNFα, IL-2 and IL-10 based on negative controls (bottom panel) and FMO controls. The percent positives are determined by subtracting the negative control values from the corresponding DENV-1 stimulated cell. All DENV-1-specific cytokine signals were statistically higher than the negative control for days 21, 28, and 42 excepting IL-10 (*p*<0.05).
(TIF)
