# Supplementary figures and images for: Primary Vaccination with Low Dose Live Dengue 1 Virus Generates a Proinflammatory, Multifunctional T Cell Response in Humans
Source: PLoS Negl Trop Dis. 2012 Jul 17;6(7):e1742. doi: 10.1371/journal.pntd.0001742 (PMC3398956; doi:10.1371/journal.pntd.0001742)

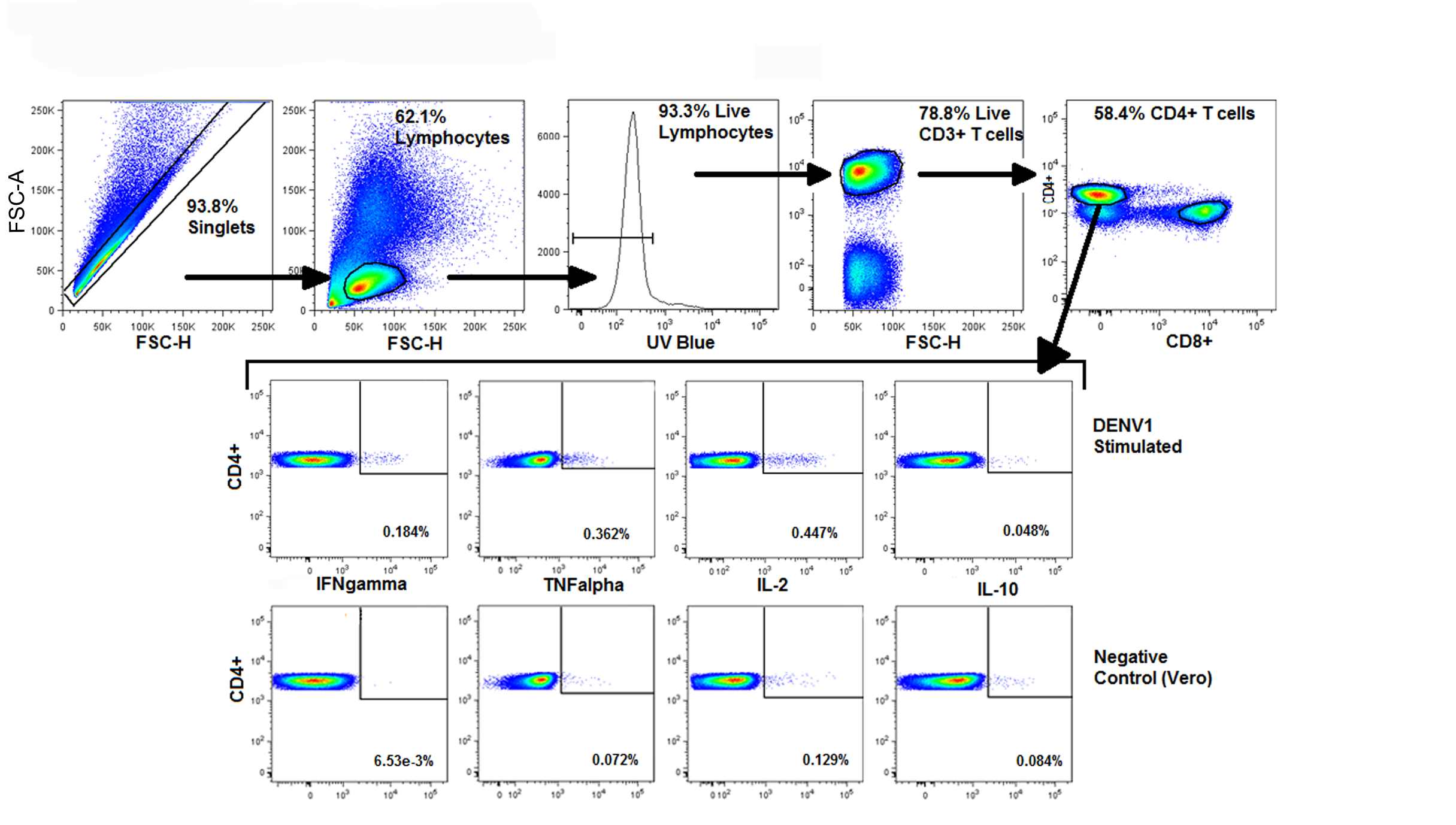

Supplement: Figure S1 — CD4+ T cells respond to DENV-1 antigen. Gating scheme and raw representative data of PBMCs stimulated with DENV-1 antigen for Subject 03 on post-vaccination day 28. Cells are initially gated on singlets followed by the lymphocyte population. CD3+ are gated from the lymphocyte population after selecting for live cells. CD4+ and CD8+ are separated from the CD3+ population. CD4+ CD8− cells are gated for IFNγ, TNFα, IL-2 and IL-10 based on negative controls (bottom panel) and FMO controls. The percent positives are determined by subtracting the negative control values from the corresponding DENV-1 stimulated cell. All DENV-1-specific cytokine signals were statistically higher than the negative control for days 21, 28, and 42 excepting IL-10 (p<0.05). (TIF) [file pntd.0001742.s001.tif]
